# Supplementary material for: CryoET structures of immature HIV Gag reveal six-helix bundle
Source: Commun Biol. 2021 Apr 16;4:481. doi: 10.1038/s42003-021-01999-1 (PMC8052356; doi:10.1038/s42003-021-01999-1)
Supplement: Supplementary file 2 — Descriptions of Additional Supplementary Files [file 42003_2021_1999_MOESM2_ESM.pdf]

Descriptions of Additional Supplementary Files

**Supplementary Movie 1**

**Description:** Movie of slices through the confidence map at 1% false discovery rate (gray) overlaid with unsharpened unsymmetrized GagΔMAT8I map (blue).
